# Measuring visual cortical oxygenation in diabetes using functional near-infrared spectroscopy

**DOI:** 10.1007/s00592-018-1200-5

**Published:** 2018-08-06

**Authors:** Ross T. Aitchison, Laura Ward, Graeme J. Kennedy, Xinhua Shu, David C. Mansfield, Uma Shahani

**Affiliations:** 1 School of Health and Life Sciences, Glasgow Caledonian University, Glasgow, UK; 2 Institute of Health and Wellbeing, University of Glasgow, Glasgow, UK; 3 Department of Ophthalmology, Inverclyde Royal Hospital, Greenock, UK

**Keywords:** Haemodynamic response, Functional near-infrared spectroscopy, Visual cortex, Diabetes, Autonomic dysfunction

## Abstract

**Aims:**

Diabetes mellitus affects about 6% of the world’s population, and the chronic complications of the disease may result in macro- and micro-vascular changes. The purpose of the current study was to shed light on visual cortical oxygenation in diabetic individuals. We then aimed to compare the haemodynamic response (HDR) to visual stimulation with glycaemic control, given the likelihood of diabetic individuals suffering from such macro- and micro-vascular insult.

**Methodology:**

Thirty participants took part in this explorative study, fifteen of whom had diabetes and fifteen of whom were non-diabetic controls. The HDR, measured as concentrations of oxyhaemoglobin [HbO] and deoxyhaemoglobin [HbR], to visual stimulation was recorded over the primary visual cortex (V1) using a dual-channel oximeter. The stimulus comprised a pattern-reversal checkerboard presented in a block design. Participants’ mean glycated haemoglobin (HbA_1c_) level (± SD) was 7.2 ± 0.6% in the diabetic group and 5.5 ± 0.4% in the non-diabetic group. Raw haemodynamic data were normalised to baseline, and the last 15 s of data from each ‘stimulus on’ and ‘stimulus off’ condition were averaged over seven duty cycles for each participant.

**Results:**

There were statistically significant differences in ∆[HbO] and ∆[HbR] to visual stimulation between diabetic and non-diabetic groups (*p* < 0.05). In the diabetic group, individuals with type 1 diabetes displayed an increased [HbO] (*p* < 0.01) and decreased [HbR] (*p* < 0.05) compared to their type 2 counterparts. There was also a linear relationship between both ∆[HbO] and ∆[HbR] as a function of HbA_1c_ level (*p* < 0.0005).

**Conclusions:**

Our findings suggest that fNIRS can be used as a quantitative measure of cortical oxygenation in diabetes. Diabetic individuals have a larger HDR to visual stimulation compared to non-diabetic individuals. This increase in ∆[HbO] and decrease in ∆[HbR] appears to be correlated with HbA_1c_ level.

## Introduction

Diabetes mellitus affects about 6% of the world’s population, and the chronic complications of the disease may result in both life- and sight-threatening macro- and micro-vascular insult. It is estimated that 415 million people worldwide are currently affected by diabetes, and this number is projected to increase to 642 million by 2040 [15]. Type 1 diabetes accounts for approximately 5–10% of cases of diabetes; type 2 diabetes accounts for the remaining 90–95% of cases [1].

Diabetes damages not only the anatomy but also the physiology of the individual. Many organs are dually innervated by the sympathetic (SNS) and parasympathetic (PNS) branches of the autonomic nervous system (ANS). It is well established that SNS hyperactivity occurs in systemic hypertension [8, 9]. However, more recent evidence has also found increased SNS activity in diabetic individuals, particularly in those with type 2 diabetes [13].

Cardiovascular autonomic neuropathy (CAN) is a common complication of diabetes that is normally associated with dysfunction of both branches of the ANS [28]. The prevalence of CAN has been found to vary [7]; however, if patients with subclinical levels of diabetic CAN are included, the prevalence may in fact exceed 90% [29]. CAN has been found to manifest first in neurones with longer axons [28]. The vagus nerve is the longest nerve of the ANS, accounting for approximately 75% of parasympathetic innervation [12]. Recent evidence has found that, in many metabolic diseases, including diabetes, decreased vagal activity may be a key underlying mechanism [23, 30].

Although the aetiology of CAN is not yet fully understood, several hypotheses have been purported. One such hypothesis is that prolonged hyperglycaemia in diabetes can lead to the formation of advanced glycation end products (AGEs) [4]. Such AGEs are seen in the glycated haemoglobin (HbA_1c_) test that is commonly performed on diabetic individuals to assess their glycaemic control. HbA_1c_ level is an indicator of blood glucose control over the preceding 8–12 weeks. For a comprehensive review of other hypotheses, please refer to Vinik et al. [30].

The haemodynamic response (HDR) is the change in the amount of haemoglobin in a portion of superficial cortex that occurs as a reflex response to increased neuronal activity. It is measured by recording changes in the quantity of haemoglobin in the primary visual cortex (V1) that occur in response to standardised visual stimulation. First described four decades ago [17], functional near-infrared spectroscopy (fNIRS) permits non-invasive measurement of the changes in cortical oxyhaemoglobin [HbO] and deoxyhaemoglobin [HbR] that comprise the HDR. Laser light sources are directed through the scalp and cranium to the cortex, and an adjacent recording ‘optode’ measures the transcranial emission of light. By selecting 687 and 828 nm as the wavelengths of recorded light, we can quantify the [HbO] and [HbR] within the volume of cortical tissue under observation, and disregard other potential coloured molecules. This technology is complimentary to functional magnetic resonance imaging (fMRI), which maps out changes in metabolites and blood flow [10]. Unlike fMRI, which requires summation of signal over sustained periods of measurement, fNIRS permits the acquisition of data in real time; though, as with visually evoked cortical electrical potentials (VEP), we do average the response over numerous repetitions of the stimulus cycle.

Checkerboards are frequently used simple stimuli that elicit a strong response over V1 [5, 21, 25, 26, 31, 33, 35]. Pattern-reversal checkerboard stimulation has been found to produce stronger cortical activity compared to static checkerboards [33].

The purpose of the current study was to shed light on visual cortical oxygenation in diabetic individuals. We then aimed to compare the HDR to visual stimulation with glycaemic control, given the likelihood of diabetic individuals suffering from macro- and micro-vascular insult.

## Methodology

### Stimulus

**Fig. 1:**
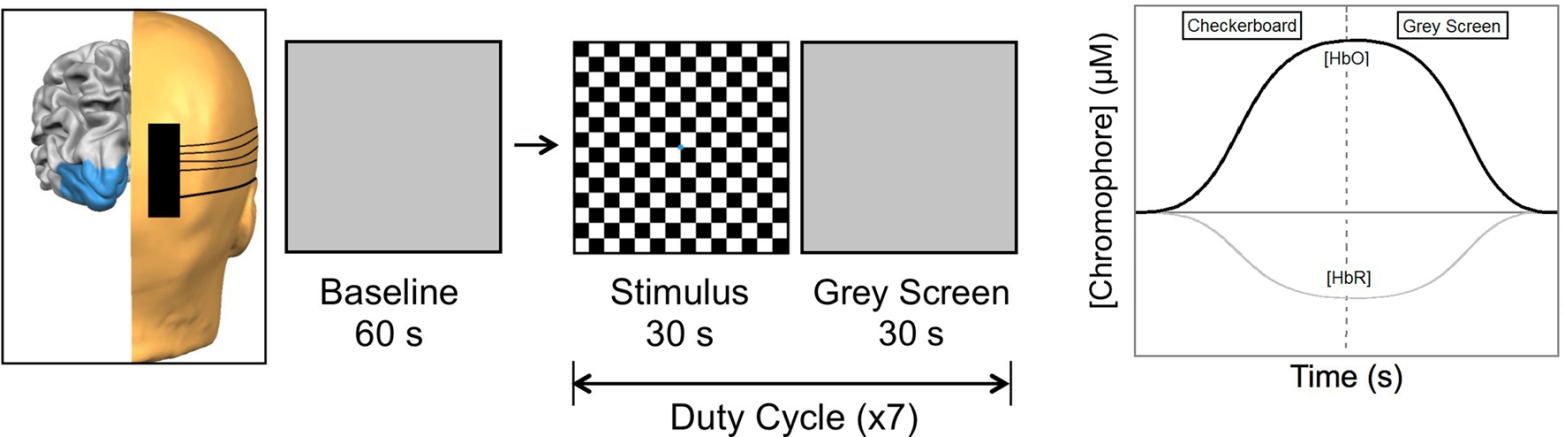
Stimulus cycle. After baseline, participants viewed a pattern-reversal checkerboard of high contrast, which was then replaced by a grey screen. This was repeated seven times. (Adapted from: Ward et al. [31])

The stimulus was a high-contrast pattern-reversal checkerboard that was viewed binocularly at 1 m. The stimulus was displayed on a 19-inch monitor with a screen resolution of 800 × 600 pixels. The check size was 30 min of arc; recent evidence has shown that high-contrast checkerboards with this check size produce the largest increase in [HbO] over the occipital cortex [31]. In accordance with the International Society for Clinical Electrophysiology of Vision (ISCEV) standards, each checkerboard had a temporal frequency of 7.5 Hz [22]. The stimulus utilised a block design. Initial baseline measures were recorded for 60 s prior to the onset of the stimulus, whereby participants viewed an isoluminant grey screen. Seven duty cycles were then presented, with each consisting of 30 s checkerboard stimulation (‘stimulus on’) and 30 s luminance-matched grey screen (‘stimulus off’) (Fig. 1).

### Apparatus

A two-channel fNIRS oximeter (OxiplexTS, ISS, Champaign, IL, USA) was used to record the HDR at occipital locations O_1_ and O_2_ over V1. The two sensors, each containing four pairs of sources and one optode, were embedded in silicone. This material had a degree of flexibility, which allowed for placement on the scalp. The sources of each sensor were located at fixed distances from the optode, ranging from 1.9 to 3.6 cm. The intensity of the near-infrared (NIR) light emitted at wavelengths 687 and 828 nm was amplitude modulated at 110 MHz, and the sampling rate was 1 Hz. The International 10–20 System of Electrode Placement was used to locate O_1_ and O_2_ [16]. Each sensor was placed vertically such that the point that was located midway between the detector and farthest emitter was placed over the region of interest. Although recordings were made over left and right hemispheres, our results consider an average V1 response (please refer to “Effect of hemisphere” below).

### Glycated haemoglobin

HbA_1c_ was measured using the A_1c_Now®+ System (PTS Diagnostics, Indianapolis, IN, USA), which used the principle of colourimetry. This device required a small 5 µl capillary blood sample, which was obtained by means of a single-use lancet. Upon application of the sample to the strip, blue micro-particles conjugated to anti-HbA_1c_ antibodies migrated along the reagent strip. The amount of blue micro-particles captured on the strip thus reflected the amount of HbA_1c_ in the sample. To determine total haemoglobin (THb) in the sample, the diluent converted THb to methaemoglobin (met-Hb). The intensity of the colour of met-Hb measured on the reagent strips was proportional to the concentration of haemoglobin in the sample. Test results were expressed as a percentage [i.e., (HbA_1c_/met-Hb) × 100].

### Participants

We recruited thirty participants, fifteen of whom had diabetes and fifteen of whom were non-diabetic controls. In the diabetic group, the mean age (± SD) was 47 ± 19 years, and the age range was 20–69 years. The male-to-female ratio was 8:7. The mean HbA_1c_ level (± SD) of the diabetic group was 7.2 ± 0.6%. The diabetic group comprised five individuals with type 1 diabetes [mean HbA_1c_ level (± SD) was 7.7 ± 0.3%] and ten individuals with type 2 diabetes [mean HbA_1c_ level (± SD) was 6.9 ± 0.6%]. The control group had a mean age (± SD) of 46 ± 23 years, and the age range was 20–71 years. The male-to-female ratio was 7:8. The mean HbA_1c_ level (± SD) of the control group was 5.5 ± 0.4%. Both groups were matched for gender [Chi square *χ*^2^ (1, *n* = 30) = 0.133, *p* = 0.715] and age (Mann–Whitney *U: U* = 95, *z* = − 0.726, *p* = 0.486). At 7.2 ± 0.6% in the diabetic group and 5.5 ± 0.4% in the non-diabetic group, the HbA_1c_ level differed significantly between groups (independent samples *t* test: *t*(28) = 8.853, *p* < 0.0005). Furthermore, the HbA_1c_ level between individuals with type 1 and type 2 diabetes was significantly different [independent samples *t* test: *t*(13) = 2.995, *p* = 0.010].

### Inclusion and exclusion criteria

All participants were aged 18 years or older, and had a best-corrected visual acuity of 0.3 logMAR or better in each eye.

Participants in the diabetic group had either type 1 or 2 diabetes mellitus, and had been diagnosed with the condition for at least 2 years. Furthermore, all diabetic participants reported no diagnosis of diabetic neuropathy (autonomic or peripheral).

Participants with any ocular disease (e.g., cataract, age-related macular degeneration) or previous damage to V1 (e.g., cerebrovascular accident, trauma) were excluded from the study.

### Statistical methods used

The raw [HbO] and [HbR] values that were recorded from each participant were processed using a custom-written MATLAB script (MathWorks Inc., Natick, MA, USA). The following analyses were performed for each participant. The experimental data were normalised to baseline, to control for within-subjects’ variance. This was done by subtracting the mean [HbO] and [HbR] recorded during the 60 s of baseline prior to stimulus onset from each individual value of [HbO] and [HbR] recorded during stimulus presentation. A moving average filter was then applied to the normalised data. The normalised data were segmented into duty cycles of ‘stimulus on’ (checkerboard) and ‘stimulus off’ condition (grey screen), with each ‘stimulus on’ or ‘stimulus off’ condition lasting 30 s. The last 15 s of data from each ‘stimulus on’ and ‘stimulus off’ condition were averaged over the seven cycles to give an average ‘stimulus on’ and ‘stimulus off’ condition for each participant.

Data were exported to SPSS Statistics 22 (IBM Corp., Armonk, NY, USA). Intraclass correlation analysis was used to assess for hemispheric differences in [HbO] and [HbR] during checkerboard and grey screen, and to assess for interhemispheric differences in ∆[HbO] and ∆[HbR] between checkerboard and grey screen. Within- and between-group analyses were conducted in SPSS Statistics 22. A three-way mixed ANOVA was run to assess how visual stimulation affected [chromophore] in diabetic and non-diabetic groups. We used a two-way mixed ANOVA to assess the ∆[chromophore] to checkerboard stimulation in diabetic and non-diabetic groups. Multivariate linear regression was used to assess the ∆[chromophore] as a function of glycaemic control. Effect sizes are reported as partial eta-squared (*η*_p_^2^) for ANOVA and the square of Pearson’s correlation (*R*^2^) for regression.

## Results

### Effect of hemisphere

We found a relationship between the HDRs of O_1_ and O_2_ during checkerboard stimulation and grey screen using intraclass correlation analysis. There was also a significant interhemispheric relationship in the change in HDR from grey screen to checkerboard (i.e., ‘stimulus on’–‘stimulus off’) (Table 1). A two-way mixed model with average measures was employed. As the responses of both hemispheres were similar, we used an average V1 response [i.e., (O_1_ + O_2_)/2].

**Table 1:** Intraclass correlation analysis between O_1_ and O_2_

	Stimulus on	Stimulus off	∆(On–off)
[HbO]	*ρ* = 0.670**	*ρ* = 0.578*	*ρ* = 0.730***
[HbR]	*ρ* = 0.550*	*ρ* = 0.541*	*ρ* = 0.612***

The intraclass correlation coefficient is denoted by *ρ* (*p ≤ 0.05; **p ≤ 0.01; ***p ≤ 0.001)

### Effect of stimulation on [chromophore]

A three-way mixed ANOVA was run to understand how checkerboard stimulation affected [chromophore] in diabetic and non-diabetic groups. The within-subjects’ factors were stimulus (‘stimulus on’ or ‘stimulus off’) and [chromophore] ([HbO] or [HbR]); the between-subjects’ factor was group (diabetic or non-diabetic). There were two outliers for [HbR] during checkerboard stimulation in the non-diabetic group whose value was greater than 1.5 times the interquartile range (> 1.5 IQR). The mean value including the outliers was not statistically significantly different to that with the outliers excluded [independent samples *t* test: *t*(26) = 0.962, *p* = 0.345]; therefore, further analysis of this ANOVA included the outliers. [Chromophore] was normally distributed in both groups during ‘stimulus on’ and ‘stimulus off’, as assessed by a Shapiro–Wilk test (*p* > 0.05).

There was a statistically significant three-way interaction between stimulus, [chromophore] and group [*F*(1,28) = 4.874, *p* = 0.036, *η*_p_^2^ = 0.148]. The simple two-way interaction between stimulus and [chromophore] was statistically significant in diabetic [*F*(1,14) = 43.394, *p* < 0.0005] and non-diabetic groups [*F*(1,14) = 28.733, *p* < 0.0005]. The simple two-way interaction between stimulus and group was statistically significant for [HbO] [*F*(1,28) = 4.426, *p* = 0.045] and [HbR] [*F*(1,28) = 5.241, *p* = 0.030].

**Fig. 2:**
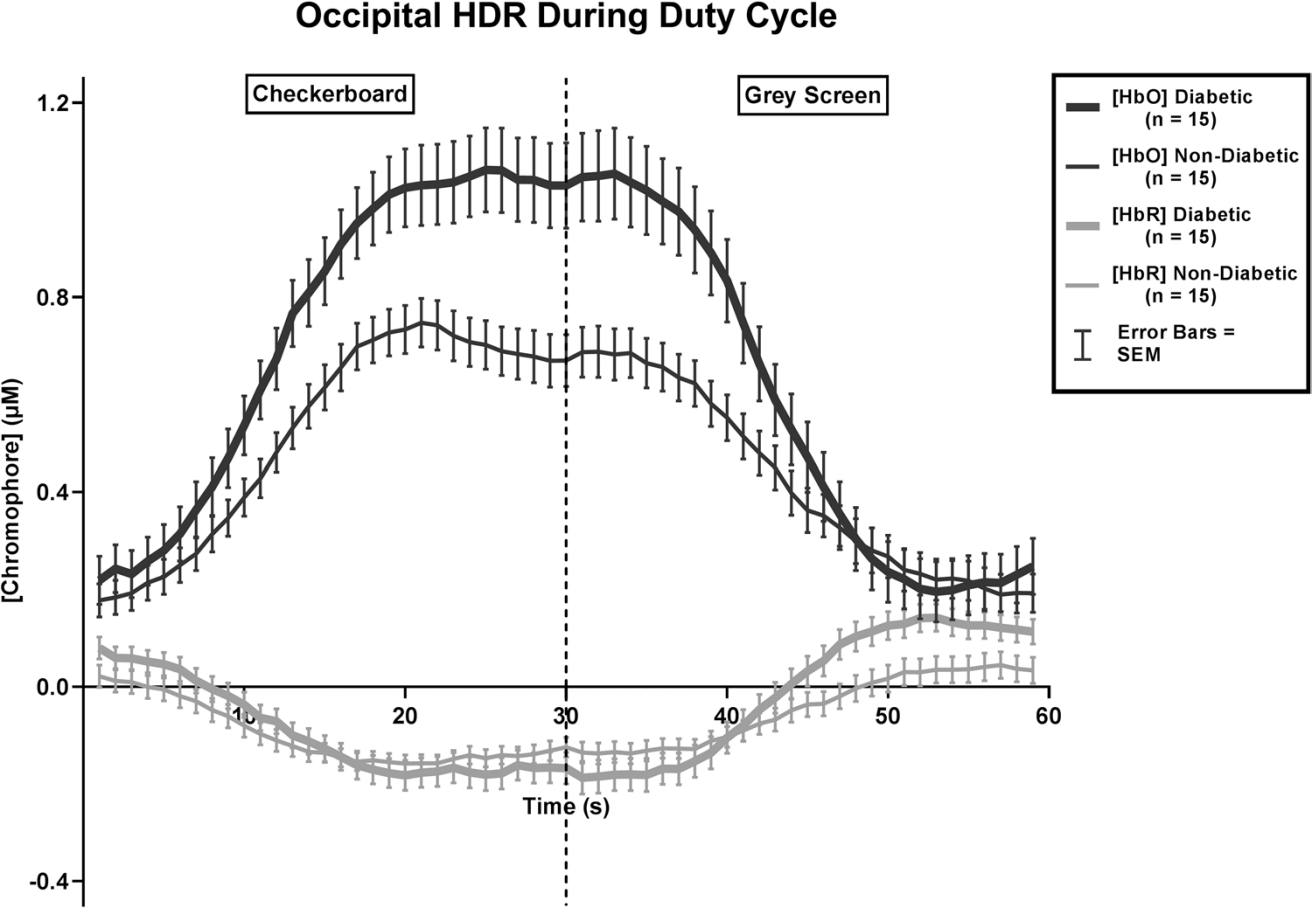
Normalised [chromophore]

There was a significant difference between [chromophore] in both diabetic [*F*(1,14) = 16.519, *p* = 0.001] and non-diabetic groups [*F*(1,14) = 38.754, *p* < 0.0005] during checkerboard stimulation (Fig. 2). However, during grey screen presentation (‘stimulus off’), [chromophore] did not differ significantly in either group.

During visual stimulation, there was a statistically significant difference in [HbO] in both diabetic [*F*(1,14) = 39.581, *p* < 0.0005] and non-diabetic groups [*F*(1,14) = 27.629, *p* < 0.0005]. In diabetic individuals, the mean (± SEM) [HbO] was 0.25 ± 0.16 µM during grey screen and 1.03 ± 0.22 µM during checkerboard. In non-diabetic individuals, [HbO] was 0.24 ± 0.14 µM during grey screen and 0.70 ± 0.11 µM during checkerboard. There was also a statistically significant difference in [HbR] in both diabetic [*F*(1,14) = 45.242, *p* < 0.0005] and non-diabetic groups [*F*(1,14) = 27.513, *p* < 0.0005] in response to visual stimulation. In diabetic individuals, the mean (± SEM) [HbR] was 0.12 ± 0.07 µM during grey screen and − 0.17 ± 0.08 µM during checkerboard, whereas in non-diabetic individuals, [HbR] was 0.02 ± 0.07 µM during grey screen and − 0.14 ± 0.05 µM during checkerboard stimulation.

### Effect of diabetes on ∆[chromophore]

We then assessed how diabetes affected the change in [chromophore] (∆[chromophore]) to checkerboard stimulation. We did this by means of a two-way mixed ANOVA. ∆[Chromophore] was calculated as the difference between [chromophore] during checkerboard stimulation and [chromophore] during grey screen (i.e., ∆[HbO] = [HbO] checkerboard—[HbO] grey screen; ∆[HbR] = [HbR] checkerboard—[HbR] grey screen). The within-subjects’ factor for this ANOVA was ∆[chromophore] (∆[HbO] or ∆[HbR]); the between-subjects’ factor was group (diabetic or non-diabetic). There were no outliers in the data, as assessed by inspection of a boxplot for values > 1.5 IQR. ∆[Chromophore] was normally distributed in both groups, as assessed by a Shapiro–Wilk test (*p* > 0.05).

There was a statistically significant two-way interaction between ∆[chromophore] and group [*F*(1,28) = 4.874, *p* = 0.036, *η*_p_^2^ = 0.148]. The main effect of ∆[chromophore] was statistically significant in both diabetic [*F*(1,14) = 43.394, *p* < 0.0005] and non-diabetic groups [*F*(1,14) = 28.733, *p* < 0.0005]. Statistical significance was also found in ∆[HbO] between diabetic and non-diabetic groups [*F*(1,28) = 4.426, *p* = 0.045]. The mean (± SEM) ∆[HbO] was 0.79 ± 0.11 µM in the diabetic group and 0.47 ± 0.11 µM in the non-diabetic group. Similar statistical significance was found in ∆[HbR] between groups [*F*(1,28) = 5.241, *p* = 0.030]. The mean (± SEM) ∆[HbR] was − 0.29 ± 0.04 µM in the diabetic group and − 0.17 ± 0.04 µM in the non-diabetic group.

### Effect of type of diabetes

We wanted to separate the effect of type of diabetes on the HDR. We assessed for this by means of a two-way mixed ANOVA in the diabetic group. The within-subjects’ factor for this ANOVA was ∆[chromophore] (∆[HbO] or ∆[HbR]); the between-subjects’ factor was type of diabetes (type 1 or type 2). There were no outliers in the data, as assessed by inspection of a boxplot for values > 1.5 IQR. ∆[Chromophore] was normally distributed in both diabetic subgroups (type 1 and type 2), as assessed by a Shapiro–Wilk test (*p* > 0.05).

A two-way statistically significant interaction was found between ∆[chromophore] and type of diabetes [*F*(1,13) = 9.882, *p* = 0.008, *η*_p_^2^ = 0.432]. The main effect of ∆[chromophore] was statistically significant for both type 1 [*F*(1,4) = 106.973, *p* < 0.0005] and type 2 subgroups [*F*(1,9) = 21.081, *p* = 0.001].

**Fig. 3:**
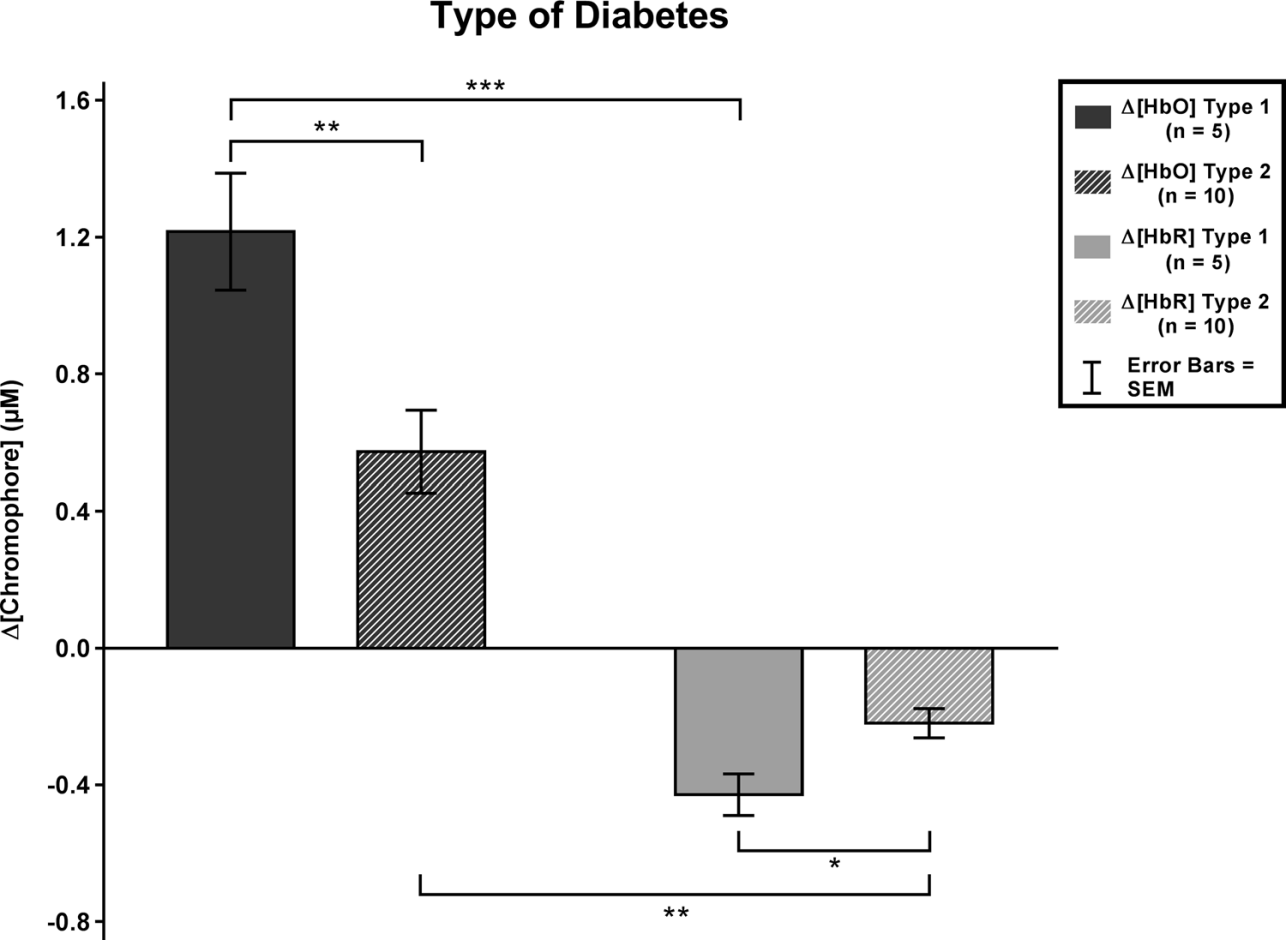
Effect of type of diabetes on ∆[chromophore] (*p ≤ 0.05; **p ≤ 0.01; ***p ≤ 0.001)

Statistically significant differences occurred between diabetic subgroups for [chromophore]. There was a statistically significant difference in ∆[HbO] between the two subgroups [*F*(1,13) = 9.414, *p* = 0.009]. The mean (± SEM) ∆[HbO] was 1.22 ± 0.17 µM in the type 1 subgroup and 0.57 ± 0.12 µM in the type 2 subgroup. This pattern was repeated for [HbR] [*F*(1,13) = 7.768, *p* = 0.015]. The mean (± SEM) ∆[HbR] was − 0.43 ± 0.06 µM in the type 1 subgroup and − 0.22 ± 0.04 µM in the type 2 subgroup (Fig. 3).

### Effect of glycaemic control

**Fig. 4:**
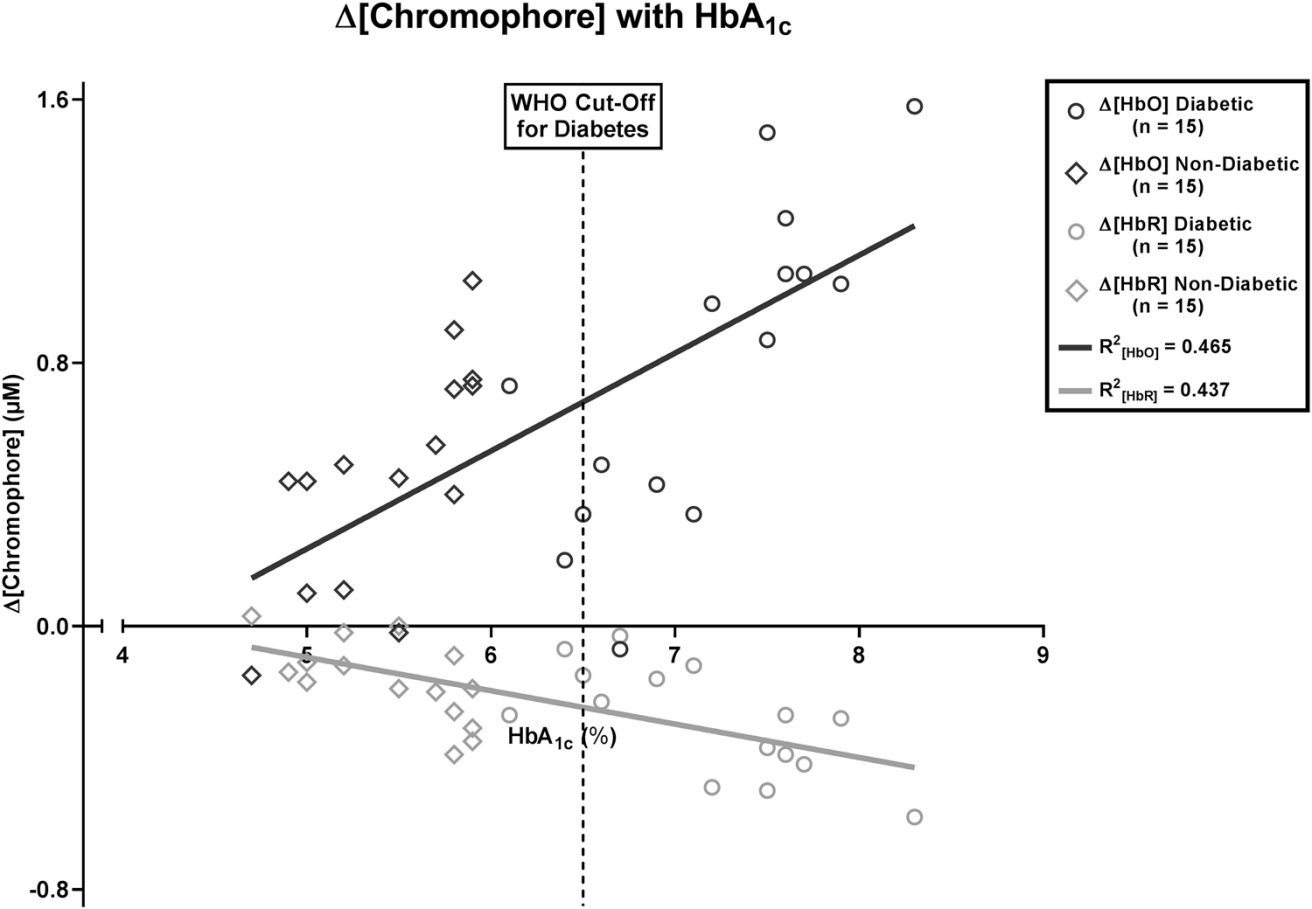
Relationship between HbA_1c_ level and ∆[chromophore]

Multivariate linear regression analysis was run to assess the effect of HbA_1c_ level on ∆[chromophore]. A scatterplot of ∆[HbO] and ∆[HbR] against HbA_1c_ was plotted for all diabetic and non-diabetic participants. Visual inspection of this plot indicated a positive linear relationship between ∆[HbO] and HbA_1c_. There was also a negative linear relationship between ∆[HbR] and HbA_1c_ (Fig. 4). Residuals were normally distributed, as assessed by visual inspection of a normal probability plot.

The prediction equations were as follows: ∆[HbO] = − 1.248 + 0.297 × HbA_1c_; ∆[HbR] = 0.415–0.102 × HbA_1c_. HbA_1c_ level statistically significantly predicted ∆[HbO] [*F*(1,28) = 24.323, *p* < 0.0005, *R*^2^ = 0.465] and ∆[HbR] [*F*(1,28) = 21.752, *p* = < 0.0005, *R*^2^ = 0.437]. HbA_1c_ thus accounted for 46.5% of the variation in ∆[HbO] and 43.7% of the variation in ∆[HbR]. Both of these are large effect sizes [6].

## Discussion

The purpose of this explorative study was to examine the differences in the HDR of diabetic individuals compared to non-diabetic individuals using fNIRS, given the likelihood of diabetic individuals suffering from macro- or micro-vascular insult. We found that the HDR to checkerboard stimulation took approximately 15 s to reach its peak, as has been reported in previous studies from this group [20, 31–34]. This was true for both diabetic and non-diabetic individuals. For our statistical analysis, only the last 15 s of data from each ‘stimulus on’ and ‘stimulus off’ condition was used. As the responses of O_1_ and O_2_ were similar, our results considered an average V1 response.

A characteristic response to visual stimulation was observed, with both diabetic and non-diabetic groups displaying an increased [HbO] and decreased [HbR] after onset of the checkerboard stimulus. With neural activity, there is an overall increase in [HbO] and decrease in [HbR], and much more oxygen is delivered than is consumed. This is a normal physiological mechanism for regional cerebral blood flow control, known as functional hyperaemia, which results in more blood flowing not only to the cortical locations where neurones are most active but also to a much larger area of cortex surrounding the active locations. Malonek and Grinvald [18] eloquently describe this phenomenon as ‘watering the entire garden for the sake of one thirsty flower’. Our results are in agreement with the findings of previous fNIRS studies [5, 20, 21, 25, 26, 31, 33, 35], all of which have found a similar functional hyperaemic response.

Diabetes is a well-established risk factor for both macro- and micro-vascular insult. The chronic complications of diabetes and vascular disease are closely intertwined in their pathogeneses. It is important for the relationship between diabetes and vascular disease to be better understood, given that the prevalence of diabetes is projected to continue to increase in the coming decades [15]. Our group analysis found that participants with diabetes had a significantly larger HDR compared to non-diabetic controls. This was reflected not only as an increase in delivery of arterial [HbO], but there was also a difference in [HbR], which is an indicator of venous return. Individuals with type 1 diabetes were found to have a larger HDR compared to those with type 2 diabetes. Moreover, we found a linear relationship between participants’ HbA_1c_ level and HDR, with there being an increase in ∆[HbO] and decrease in ∆[HbR] as a function of their glycaemic control. HbA_1c_ accounted for 46.5% of the variation in ∆[HbO] and 43.7% of the variation in ∆[HbR], both of which are large effect sizes [6]. However, our results from diabetic individuals with chronic hyperglycaemia differ from those of an fMRI study, in which the authors found no substantial effect on activation of the occipital cortex in non-diabetic individuals with induced acute hyperglycaemia [11]. Perhaps the normal functioning of the ANS of non-diabetic individuals may be responsible for this. The ANS of diabetic individuals is generally chronically compromised; therefore, a short period of induced hyperglycaemia in non-diabetic individuals would not necessarily mimic a diabetic response.

There are two hypotheses for this apparently linear relationship between HDR and glycaemic control, both of which involve ANS dysfunction; these are over-action of the SNS and under-action of the PNS. It is well established that SNS hyperactivity occurs in systemic hypertension [8, 9] and is involved in the pathogenesis of the macro- and micro-vascular damage that ensues [19]. Systemic hypertension coexists in 80% of cases of type 2 diabetes and in 25% of cases of type 1 diabetes [3]. The presence of systemic hypertension in diabetes is known to augment macro- and micro-vascular insult [14]; even in normotensive individuals with type 2 diabetes, SNS activity is higher than in non-diabetic individuals [13]. Although SNS over-action may help us to understand why en bloc diabetic individuals had a higher HDR compared to their non-diabetic counterparts, it does not account for the larger HDR found in individuals with type 1 diabetes compared to those with type 2 diabetes.

Another hypothesis is that higher than normal arterial blood flow to the visual areas in response to visual stimulation could be an indicator of CAN. Poor glycaemic control is a major risk factor for the development of CAN in diabetes [36]. Although it is a disorder associated with dysfunction of both branches of the ANS, CAN has been found to manifest first in neurones with longer axons [28]. The vagus nerve is the longest nerve of the ANS, accounting for approximately 75% of parasympathetic innervation in the body [12]. Recent evidence has found that, in many metabolic diseases, including diabetes, decreased vagal activity may be a key underlying mechanism [30]. Early in the course of the disease, CAN tends to be associated with PNS denervation, and the SNS branch predominates with reduced opposition from the PNS. This alters the SNS–PNS tone, and a compensatory increase in the cardiac sympathetic tone ensues [24, 27]. Although we cannot prove causality, it can be hypothesised that the increased HDR as a function of glycaemic control may be due to ANS dysfunction, either by over-action of the SNS or under-action of the PNS.

Most diabetic individuals in this study had relatively good glycaemic control, in that the highest HbA_1c_ level recorded from any participant was 8.3%. Future studies should aim to include diabetic participants with elevated or severely elevated HbA_1c_ levels. This would be with a view to determining: (1) whether the HDR continues to increase linearly as a function of glycaemic control, which may suggest over-action of the SNS; (2) whether the HDR reaches a plateau at given HbA_1c_ level; (3) whether the HDR reduces as the SNS branch also becomes affected in the course of CAN progression. In addition, other measures of autonomic activity should be included, such as heart rate and blood pressure. Heart rate variability would be another such measure, as it has been found to be directly linked to parasympathetic vagal activity [2]. Furthermore, in order for the results to be generalisable to the population, future studies should include many more participants from various centres.

In conclusion, our findings suggest that fNIRS can be used as a quantitative technique to measure cerebral oxygenation in diabetes. We have found that diabetic individuals have a larger HDR (reflected as increased ∆[HbO] and decreased ∆[HbR] to visual stimulation) compared to non-diabetic individuals. These ∆[HbO] and ∆[HbR] appear to be correlated with HbA_1c_ level. The apparently linear relationship between HDR and glycaemic control may be due to ANS dysfunction.
